# Nurses’ role in diabetic foot prevention and care; a review

**DOI:** 10.1186/2251-6581-11-24

**Published:** 2012-11-21

**Authors:** M Aalaa, O Tabatabaei Malazy, M Sanjari, M Peimani, MR Mohajeri-Tehrani

**Affiliations:** 1 Endocrinology and Metabolism Research Center, Tehran University of Medical Sciences, Tehran, Iran

**Keywords:** Diabetes, Diabetic foot, Nurse

## Abstract

Diabetes as one of Non-communicable diseases has allocated a large proportion of cost, time and human resources of health systems. Now, due to changes in lifestyle and industrial process, incidence of diabetes and its complications have been increased. Accordingly diabetic foot considered as a common complication of diabetes.

Nurses are health care providers who actively involved in prevention and early detection of diabetes and its complications. The nurses’ role could be in health care, health, community education, health systems management, patient care and improving the quality of life.

Diabetes Nurses play their educating role in the field of prevention of diabetic foot, foot care and preventing from foot injury. In care dimension, nurses responsible for early detection of any changes in skin and foot sensation, foot care, dressing and apply novel technology.

In the area of rehabilitation, help patient sufferings from diabetic foot ulcer or amputation, to have movement are diabetes nurse’s duties.

Consequently, nurses need to attend in special training to use the latest instructions of diabetic foot care in order that provides the effective services to facilitate promote diabetic patients health.

## Background

According to the report of World Health Organization (WHO) the number of diabetic patients in 2000 reached to 171 million [1] and was predicted to increase 380 million by 2025. So, at now in most countries diabetes is becoming as an epidemic disorder. There exist evidence demonstrating the significant consequences of the disease on both health care providers and the community as a whole [2,3]. Solving this problem requires close collaboration among health system and people; develop national and international strategies and interaction with other health team members. By this approach, providing adequate and effective health services are necessary for patients and their families Also, improving the quality of nurses’ clinical performance can lead to changes in client and patient societies [4].

The novel treatments can lead to increase longevity of diabetic patients and the risk of chronic complications such as eye involvement, renal, cardiovascular and diabetic foot and also cause to impose heavy economic burden on the health system [5]. Among diabetes’ complications, different types of foot problems such as ulcers and infections are common and it has shown an increasing trend in the past decade [6]. Diabetic foot is defined by WHO as foot in diabetics with neurologic disorders, some degree of vascular involvement with or without metabolic complications of diabetes in lower extremity and prone to infection, scarring, with or without deep tissue damage [7]. Some studies have shown 15 percent of diabetic patients will be suffering from diabetic foot ulcer during their lifetime [8-14]. Diabetic foot ulcer is the most general cause of hospitalization in diabetic patients [15]. On the other hand, these ulcers can lead to infection, gangrene, amputation and even death if the necessary care is not provided [16]. In addition, lower extremity amputation is associated with prolonged hospitalization and rehabilitation and also is required to home care and social support [17]. Overall, the rate of lower limb amputation in diabetic patients is 10–30 times higher than non diabetics [18,19]. The studies showed that every 30 seconds one leg is amputated due to diabetes in the world [20]. In the first two years after amputation, there is a 50 percent risk of re-amputation [21] and three years after lower limb amputation, 50% of patients may be dead [22]. The prevalence of diabetic foot in Iran was estimated at 3% in 2001 [23].

It should be noted that, care and treatment of diabetic foot is expensive all around the world. In developed countries, more than 5% of diabetics have foot ulcers and 20% of total health care resources spent on care of the diabetic foot in these countries. In other words, the cost of treating a diabetic foot ulcer is 7000–10000 US $, and when the complicated and need to amputation, this cost will be increased by 65,000 US $ [24]. Whereas, in developing countries not only diabetic foot and its complications are more common, but also even sometimes up to 40% of health care resources are unique to this disease [25]. Besides, the burden of this disease is high. The study was conducted in 2001 for estimation burden of diabetes in Iran; the burden of diabetic foot was estimated at 5848 and by adding the burden of neuropathic diabetic foot was received up to 40,000 [26]. It should be considered that the burden of diabetic foot related neuropathy was two folds than the burden of diabetic retinopathy or nephropathy currently [26].

The development of diabetic foot ulcers results from several factors. These factors can increase the risk of foot ulcer and cause detachment in the skin or impairment in the wound healing. Peripheral neuropathy can cause excessive pressure on some points of the feet and consequently, ischemia can increase the susceptibility to ulceration by impairment in peripheral vascular. In addition, other factors such as poor vision, limited joint movement, inadequate foot coverage and shoes can be susceptive to ulceration in diabetics [27-30]. The most important point is that 85% of diabetic foot amputations are preventable with appropriate care and education [31]. Ideal management for prevention and treatment of diabetic foot is as follow: regular perception of foot, determine at risk foot, education to patient and health staff, appropriate foot coverage, and early treatment of foot problems [32].

According to the protocol recommended by the American Diabetes Association (ADA), one of preventive tactic in diabetes care is multidisciplinary team approach that its advantages are shown in several studies [33,34]. The multidisciplinary team can reduces amputation rates [35-41], prevent diabetes’ complications and save costs as 1,824 U.S. $ in the standard treatment group and 1,127 U.S. $ in intervention group [42]. The result of study was shown by multidisciplinary team approach the two-year incidence of diabetic foot ulcers was 30% and 58%, respectively in high risk patients and in group under treatment with standard therapy [43]. The members of team for diabetic foot care usually consists of general practitioner, nurse, educator, orthotic, and podiatrists and some consultants; vascular surgeon, infection disease specialist, dermatologist, endocrinologist, dietitian, orthopedic and also it is necessary the access to centers and home care services [44,45]. Although all team members have influence on reduction the incidence of foot ulcer and amputation [46], however, the role of nurse and podiatrists are essential [47].

This study investigated the assessment role of the nurse as a member of team of diabetes care, for prevention and control of diabetic foot in the three areas; education, care and rehabilitation.

### Goals of nursing intervention in diabetic foot care

Improvement of patient care and health services are one of the most important challenges for nurses. According to World Health Organization, nurses are one of the largest health groups in the world who are involved in different levels of health.

Obviously, there are several reasons for the presence of nurses in the health care team, but in general, the four major goals are included health promotion, prevention of diseases, patients care, and simplify patients’ compliance. To achieve these goals, nurses can play different roles. There are seven main roles for nurses including: 1. providing health care, 2. care connector, 3. educator, 4. consultant, 5. leader, 6. researcher, 7. supporting the rights of patients [48].

Nurses combine science and art to provide health services and seek to eliminate physical, emotional, mental, social-cultural and spiritual patient needs. Since patients care is the first duty of nurses, so that they play an important role in the care of diabetes in developed countries, and diabetes nursing is divided into several categories, including nurse practitioner, clinical nurse specialist, diabetes nurse, generalist nurse and each of them has clear duties. For example, nurse practitioner focuses on health promotion and disease prevention activities including patient education and consulting [49].

It is obvious that with the increasing prevalence of diabetes and its complications, there is undeniable need to train nurse specialist in this field. The diabetic foot is so important to such an extent that was considered as one of the main objectives of the Healthy People 2010 to reduce the incidence of foot ulceration and amputation in diabetic patients. So it was targeted a 55% reduction in amputations and an increase of nearly 75% in diabetic foot examinations [50,51].

### Nurse’s role in education

It has been observed that nurses have an effective role in prevention of foot ulcers and lower limb amputation by educational interventions, screening high-risk people and providing health care [52]. It is necessary for all diabetic patients, especially patients at risk for foot ulcers, to be familiar with the basics of foot care. Several studies suggest that patient education about foot care is effective in prevention of diabetic foot ulcers [53-55]. Nurses can teach patients how to perform physical examination and take care of their feet on a daily basis [56]. For instance, nurses can encourage patients to carry out a series of simple rules in order to help prevent foot ulcers or recurrence, such as checking the shoes before wearing, keeping feet clean and continuing care of the skin and nails. Training about choosing the right shoes is essential as well [57].

Diabetic foot care education programs have been proposed [58] considering the consequence of continuing educational programs, which are detailed in Table 1[59-61]. However, the nurse educators can evaluate patient requirements and design a particular educational program for each of patients and their families [33]. Nurses can facilitate active participation of patients and family members in care and they can also teach patients about the importance of regular visits to the clinic, blood tests at specified intervals and the primary principle of diabetes care and prevention of its complication.

**Table 1:** The basic principles of foot care in clinic and home visit

	
• To Examine feet daily for discoloration, swelling, skin cracks, pain or numbness	
• Use the self help methods to help foot examination such as using mirrors	
• Foot hygiene (daily washing, followed by drying feet carefully, especially between the fingers)	
• Controlling water temperature before washing foot	
• To avoid going barefoot or wearing shoes without socks	
• To choose shoes that are precisely in size. The best time for buying shoes is in the afternoon.	
• Cutting the fingernails directly	
• To avoid manipulation of foot lesions such as corn	
• To keep wet the dry surfaces of foot by moisturizing creams except between the fingers	
• To ask for help if reduction of the visual acuity.	

Moreover, since hyperglycemia is a modifiable risk factor in diabetic neuropathy, appropriate blood sugar control is paramount to reducing neuropathy and improving patients’ quality of life. Hence, patients with poor control of blood glucose should be given special attention for practicable educations [62]. This emphasizes the importance of nurses’ role to develop a comprehensive educational program. Besides learning the suitable life style, patients should be trained according to the severity of diabetic foot problems [47].

Considering the above points, education of health care provider is a crucial issue. In addition, most of the time, they lack the knowledge of effective treatments. In other words, along with patient education, diabetic foot team members and nurses as a key member of them must be trained. The main goal of it is to increase staff awareness of the diabetic foot ulcer risks and improve their skills in examination and treatment of diabetic foot [41].

### Nurses’ role in care

#### Examination and screening

Peripheral neuropathy, peripheral vascular disease and infection are three major factors for diabetic foot ulcer that can lead to gangrene and amputation [63]. However, peripheral neuropathy is solely responsible for more than 80% of foot ulcers in diabetic patients. This not only is important for neurological examination as the first criterion for screening patients at risk for foot ulcers [23], but also is indirectly emphasized on nurse’s role in performing a diabetic foot examination with monofilament and collaboration with other diabetic foot team members.

Nurses who specialize in foot care are involved in the early stages of care and treatment [64]. Nurses’ role in diabetic foot care includes foot examination, wound dressing [33], also encouraged patients and families to appropriate care and follow-up visits regularly [65,66]. The primary goal of screening is early detection of diabetic foot problems, identifying those at risk and planning to reduce the risk of ulcers [67].

Diabetic foot examination should be part of all visits. Nurses should ask patients to remove their shoes and socks [68-70], and then examine their feet in order to screen patients at high risk and report to other members of the multidisciplinary diabetic foot team [71].

In a diabetic foot specialty clinic, nurses may access vascular status with an ankle brachial index (ABI) and toe pressure. Moreover, pedography system and thermometer are used to assess foot sole pressure and foot temperature [72,73] so the severity of foot problems and being at risk of diabetic ulcers will be identified.

### Nurse cooperation in the diabetic foot treatment

Another part of duties that a nurse provides to produce excellent diabetic foot care should be the complementary care such as selection an appropriate dressing according to the type of ulcers. Selection dressing depending on the type of wound which is wet or dry is important since dressings, while keeping clean the wound and maintain the wound moisture, help to debridement and reduce the number of bacteria [74,75]. Regarding the variety of novel dressing, awareness and knowledge of nurses in this field needs to be improved.

### Nursing role in diabetic foot care at home

Diabetic patients follow up at specified intervals is part of the care plan which should be considered first. Accordingly, all diabetics should be referred to the diabetes clinic in order to have been evaluated for diagnostic and comprehensive foot care every year [34].

Daily foot care for some diabetic patients, especially patients with limited vision due to diabetes and other chronic diseases are difficult because they could not be able to evaluate their feet.

Peripheral vascular disease, decreased foot sensation in combination with delay wound healing cause difficulty in foot care. These complications should be evaluated by nurses in both clinic and home visit.

Diabetic foot nurses while examining the patient’s feet in clinic or at home should have completed the initial patient evaluation list and examined the limb movement, health, moisture, color, temperature, edema, pain and sensation of the foot [48].

### Nursing role in rehabilitation

One of the nurses’ duties is helping patients with diabetic foot ulcers to have the movement. This is vital especially for patients who have lost their foot. Nurses should encourage and teach patients to use assistive devices [76-78]. Accordingly, nurses should be identifying different types of devices and its applications so that introduce to the patients based on patient conditions to maintain their mobility.

For example, duties of a diabetic foot nurse in this field include introduction, training and participation of patients in the make use of devices such as canes, walkers and wheelchair (which completely remove the pressure on the limb) along with the aids such as shoes, boots, the Scottish stone, full contact plaster, plaster walker are an effective methods for removing pressure of the foot [79].

## Discussion

Diabetic foot as the most common cause of hospitalization in diabetic patients is one of health system concerns. So that most of the time of diabetes healthcare providers is allocated to the prevention and diagnosis of diabetic foot complications. In this regard, nurses as members of the diabetes care team not only need to be play their role in health care, public education, health system management, patient care and improving the quality of life, but also must attend in special training to use the latest instructions of diabetic foot care in order that provides the effective services to facilitate promote diabetic patients health.

In our country, despite the increased number of diabetic patients, the training of specialist nurses such as diabetes or diabetic foot specialist nurses has not been considered effectively. It seems that developing short term training courses for nurses, use of diabetic foot clinical guidelines and algorithms in clinics and hospitals along with continues training about novel approaches in diabetic foot care could be temporarily increased the focus on diabetes and foot care. Moreover the wide spectrum of programs includes the Master of Sciences in Nursing for developing diabetes specialist nurse and development of electronic health can be diminished this global problem.

In this regard, Endocrinology and Metabolism Research Institution of Tehran University of Medical Sciences developed the clinical guideline of diabetic foot, translated the clinical care of diabetic foot, developed the diabetic foot section of the virtual clinic for diabetes education [80] designed diabetic foot website [81] and also has established the network of diabetic foot clinics in order to these educational resources used by diabetic foot care team including nurses.
